# Complete Genome Sequence of *Enterobacter* sp. Strain R4-368, an Endophytic N-Fixing Gammaproteobacterium Isolated from Surface-Sterilized Roots of *Jatropha curcas* L.

**DOI:** 10.1128/genomeA.00544-13

**Published:** 2013-08-01

**Authors:** Munusamy Madhaiyan, Ni Peng, Lianghui Ji

**Affiliations:** Biomaterials and Biocatalysts Group, Temasek Life Sciences Laboratory, National University of Singapore, Singapore

## Abstract

*Enterobacter* sp. strain R4-368 is one of the few characterized *Jatropha* endophytic diazotrophic bacteria and was isolated from surface-sterilized roots. This bacterium shows strong growth-promoting effects, being able to increase plant biomass and seed yields. *Enterobacter* sp. R4-368 is the second fully sequenced diazotrophic *Enterobacter* species. The sequence information shall facilitate the elucidation of the molecular mechanisms of plant growth promotion, nitrogen fixation in nonlegume plant species, and evolution of biological nitrogen fixation systems.

## GENOME ANNOUNCEMENT

*Jatropha curcas* is a biofuel crop targeted to marginal land where soil nutrients are low (1–3); the requirement for nitrogen fertilizer is higher than for other crops. From surfaced-sterilized root tissues, we have identified an *Enterobacter* sp. strain R4-368 that strongly fixes nitrogen *in vivo* and improves the growth and seed productivity of *J. curcas* (4). The 16S rRNA gene sequence (1,478 bp) of strain R4-368 shares 99% identity with that of *Enterobacter cloacae* subsp. *dissolvens* SDM, which lacks nitrogen-fixation genes (5). To date, 10 *Enterobacter* full-genome sequences or whole-genome shotgun scaffolds have been published (6–12). However, *Enterobacter radicincitans* DSM16656^T^, isolated from the phyllosphere of winter wheat, is the only diazotroph that has been shown to promote the growth of several plant species (9).

The genomic DNA sample of strain R4-368 was sequenced on a FLX Titanium platform at Macrogen, Inc. (Republic of Korea). A shotgun and 3-kb paired-end library sequencing reads, each with about 30× sequence coverage, were assembled using the GS De Novo assembler (v 2.6). Assembly of both libraries yielded 10 scaffolds covering 5,210,394 bases, with an average size of 521,039 bases, an *N*_50_ size of 848,763 bases, and the largest scaffold size of 1,996,153 bases. The small sequence gaps were filled by sequencing PCR products by using the BigDye Terminator v3.1 cycle sequencing method. Results of a BLAST search suggested that scaffold 7 (116,007 bases) is a plasmid, as it shares high sequence homology with the *E. cloacae* plasmid pEC-IMPQ (GenBank accession number EU855788). This assumption was confirmed by extensive sequencing of PCR products spanning two open ends. Scaffold 10 (3,239 bases) is apparently an rRNA gene consensus sequence encoding the 23S rRNA and 5S rRNA. The remaining 8 scaffolds were tentatively plotted to the genome sequence of *Enterobacter* sp. 638 (13). The sequence gaps and the joining order of the scaffolds were rigorously confirmed by sequencing of long-range PCR and inverse PCR products using the Expand Long PCR kit (Roche) with at least one primer targeting the rRNA flanking region and the other in a nonconserved region in the spacer region in the rRNA genes. The assembled genome of strain R4-368 comprises a single circular chromosome of 5,039,027 bp, with a G+C content of 54.0% and one plasmid, designated pENT01 (116,007 bp), having an overall G+C content of 52.8%. The assembled genome sequence was annotated using the NCBI Prokaryotic Genome Annotation Pipeline (PGAAP) (http://www.ncbi.nlm.nih.gov/genome/annotation_prok/). A total of 4,948 genes were assigned though the PGAAP and categorized into 4,827 CDS, 7 pseudogenes, 27 rRNAs (5S, 16S, and 23S), 87 tRNAs, and 6 frameshifted genes.

Several gene clusters related to plant growth promotion were found in the genome, including a complete set of genes for nitrogen fixation and synthesis and efflux of volatile compounds (acetoin and 2,3-butanediol), auxin, and siderophore.

### Nucleotide sequence accession numbers. 

The genome sequences of the chromosome and plasmid of *Enterobacter* sp. strain R4-368 have been deposited in GenBank under the accession numbers CP005991 and CP005992, respectively.
